# Supplementary material for: Glacial lake outburst floods threaten millions globally
Source: Nat Commun. 2023 Feb 7;14:487. doi: 10.1038/s41467-023-36033-x (PMC9905510; doi:10.1038/s41467-023-36033-x)
Supplement: Supplementary file 1 — Supplementary Information [file 41467_2023_36033_MOESM1_ESM.pdf]

Supplementary Information for Glacial Lake outburst floods threaten millions globally.

Taylor et al.

This PDF contains:

Supplementary Figures:

Supplementary Figure 1. Number and area of glacial lakes per region as of 2020.

Supplementary Figure 2. Spatial distribution of exposure to GLOF for each nation grouped by mountain range.

Supplementary Figure 3. Comparisons of GLOF research items per regions and calculated 2020 GLOF danger.

Supplementary Figure 4. Mountain range totals for indicators used to the calculate social vulnerability index.

Supplementary Figure 5. Workflow detailing the extraction of population exposure to GLOFs.

Supplementary Figure 6. Top 3 most dangerous catchments in this study.

Supplementary Tables:

Supplementary Table 1: Top 50 basins in terms of GLOF danger as of 2020.

Supplementary Table 2: Raw values used to calculate the Social Vulnerability Index for the vulnerability proxy. Values are given as percentages of the total national population.

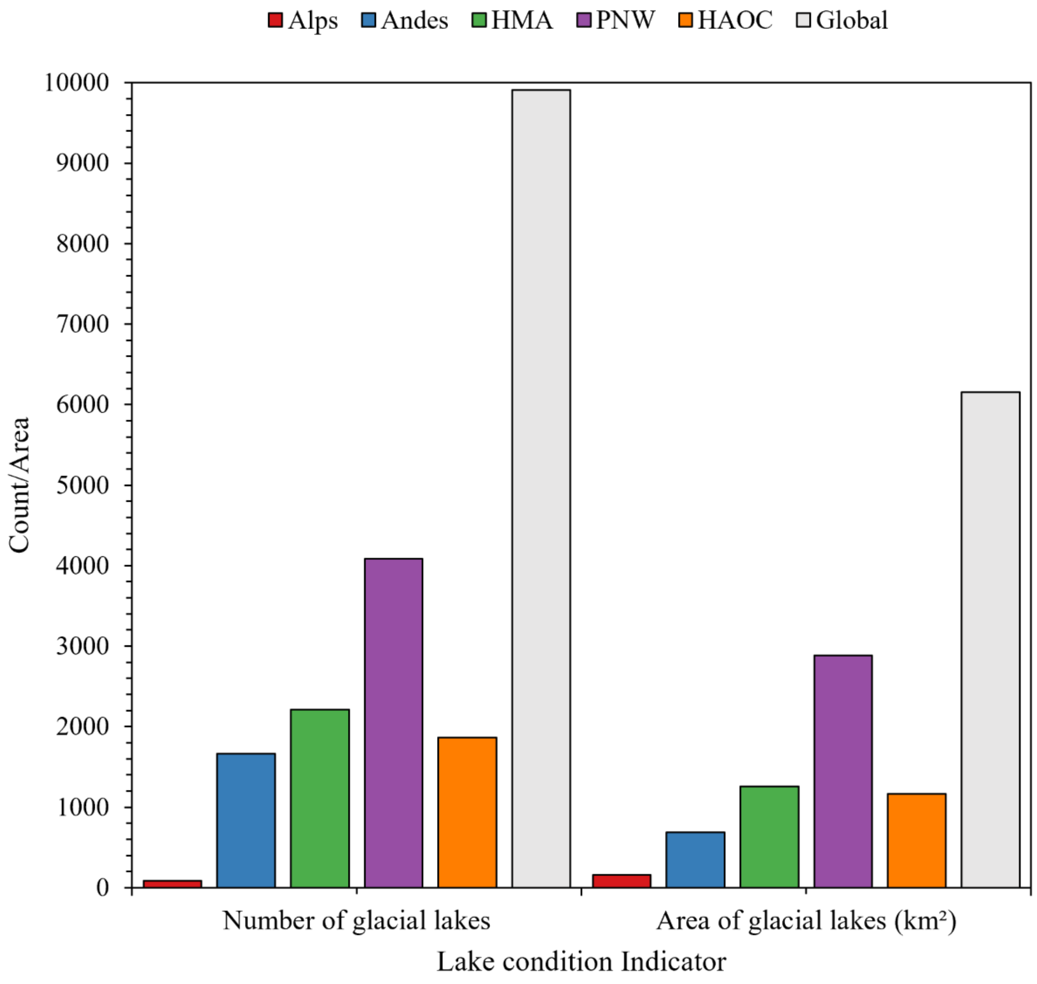

ID	Number of glacial lakes	Area of glacial lakes (km^2)	Normalised Condition Score
Alps	87	159.51	0.041
Andes	1662	686.87	0.334
HMA	2211	1256.09	0.405
PNW	4083	2884.05	1.000
HAOC	1862	1166.09	0.447
**Global**	9905	6152.60	

Supplementary figure 1: GLOF lake conditions. Number and area of glacial lakes and normalised lake condition score based on both number and area of glacial lakes for each mountain range in the study.

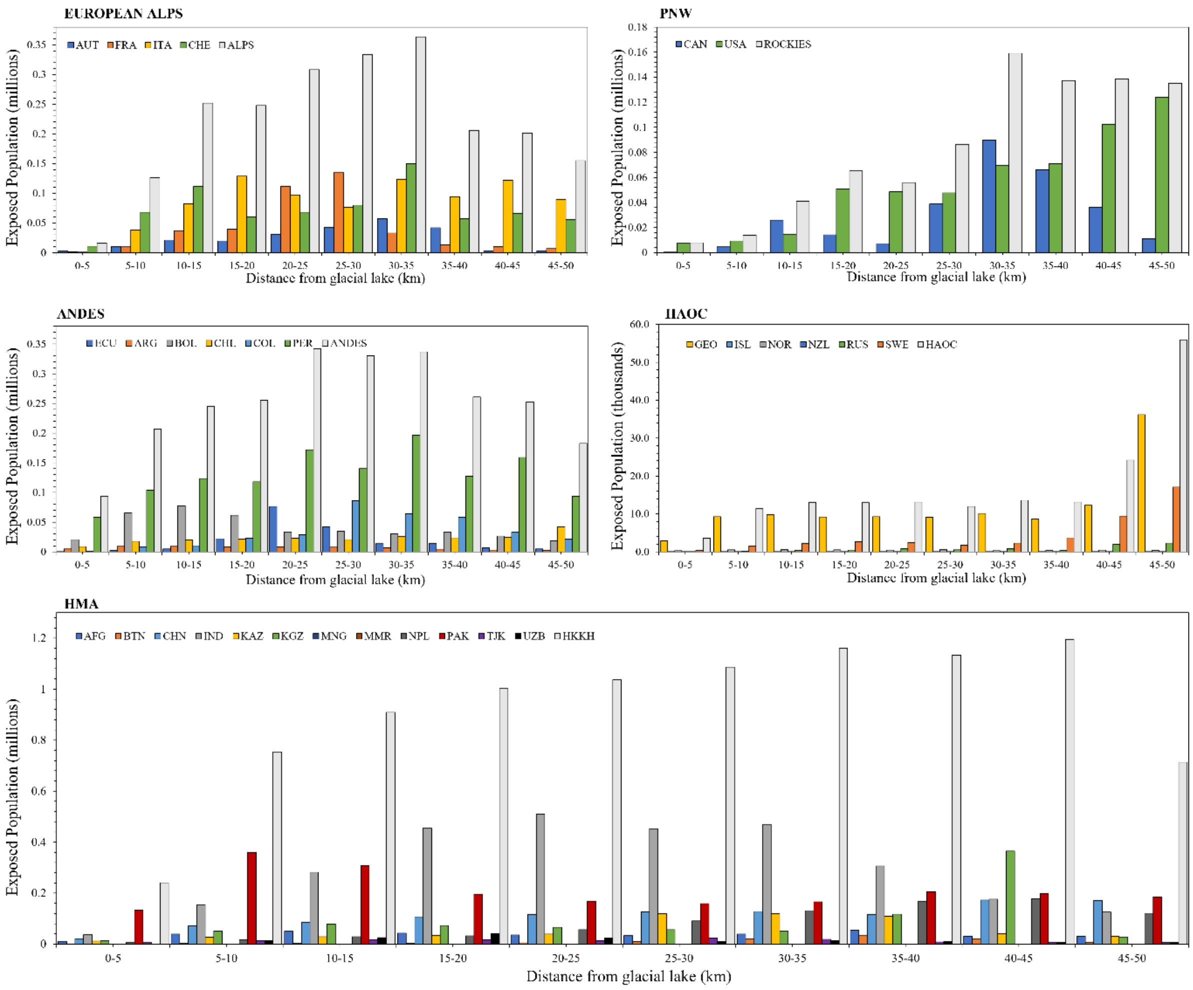

Supplementary figure 2: National spatial distribution of exposure. Spatial distribution of exposure to GLOF within 1 km of likely GLOF runout tracks up to 50 km from a glacial lake, at 5 km intervals for each mountain range.

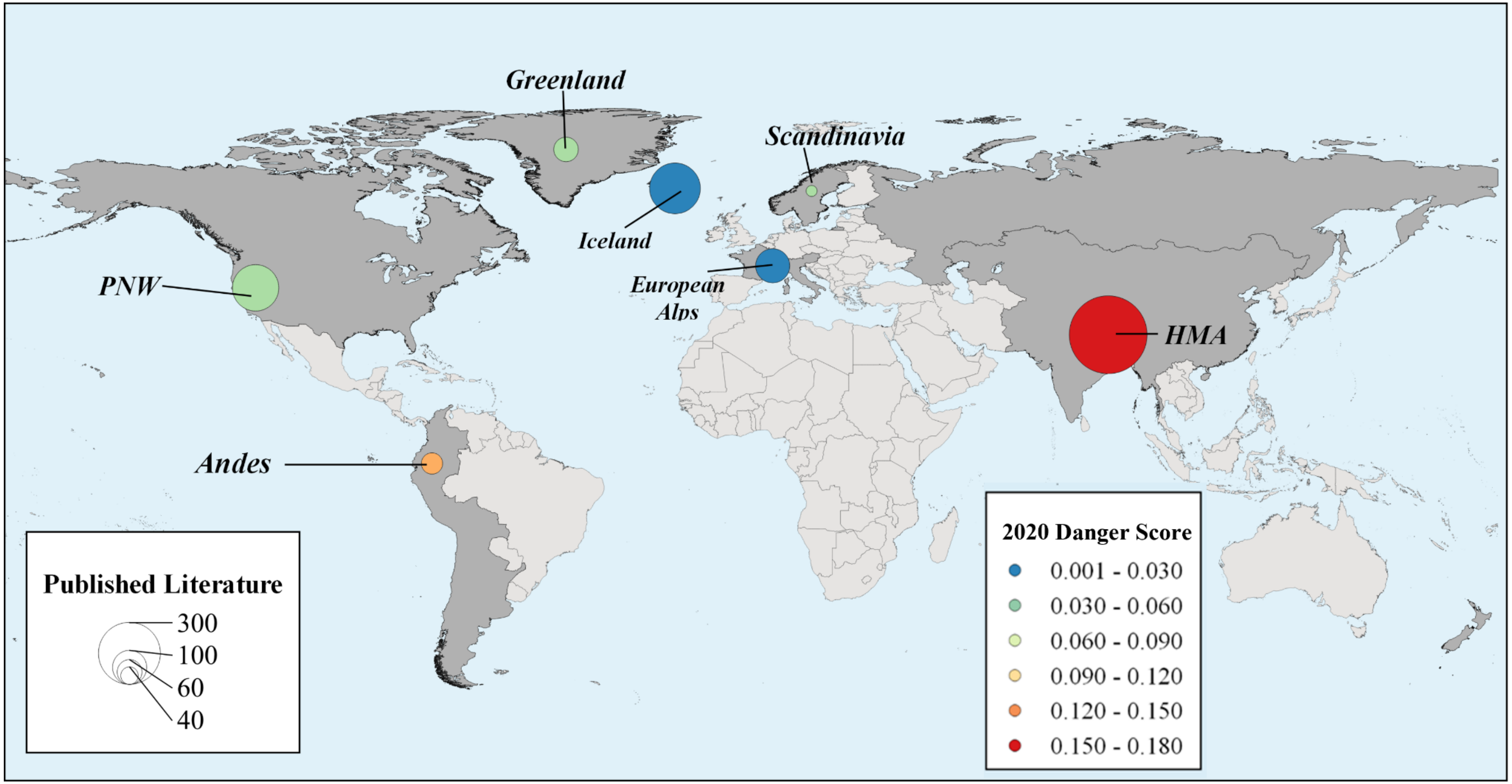

Supplementary figure 3: GLOF research. Number of GLOF research items per regions compared to calculated 2020 GLOF danger. Generally, regions with lower danger (e.g. Iceland, PNW) have been highly studied, whereas the high danger regions (e.g. the Andes) have fewer studies. Regions in grey indicate those containing glacial lakes.

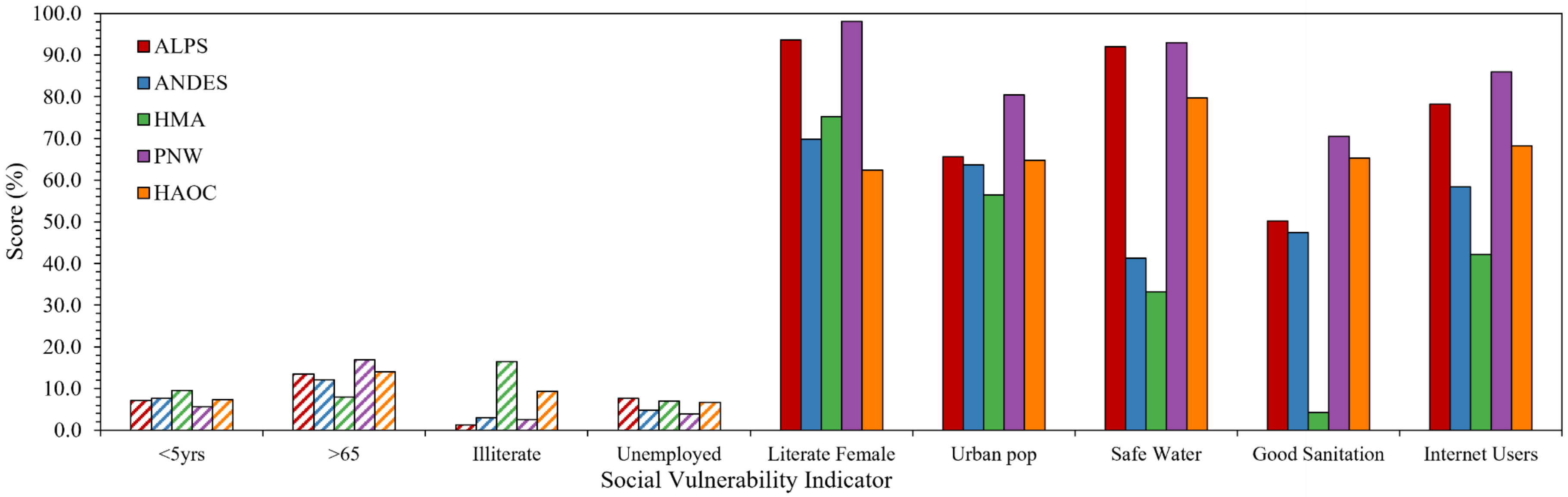

Supplementary figure 4: Social Vulnerability Index. Mountain range totals for indicators used to the calculate social vulnerability index used in this study. Dashed bars show factors that increase vulnerability to GLOFs; percentage population < 5 years of age, percentage population > 65 years of age, percentage population illiterate and percentage population unemployed. Solid bars show factors that decrease vulnerability to GLOF; percentage female population with some literacy, percentage urban population, percentage population with access to safe drinking water, percentage population with access to good sanitation and percentage population with access to internet.

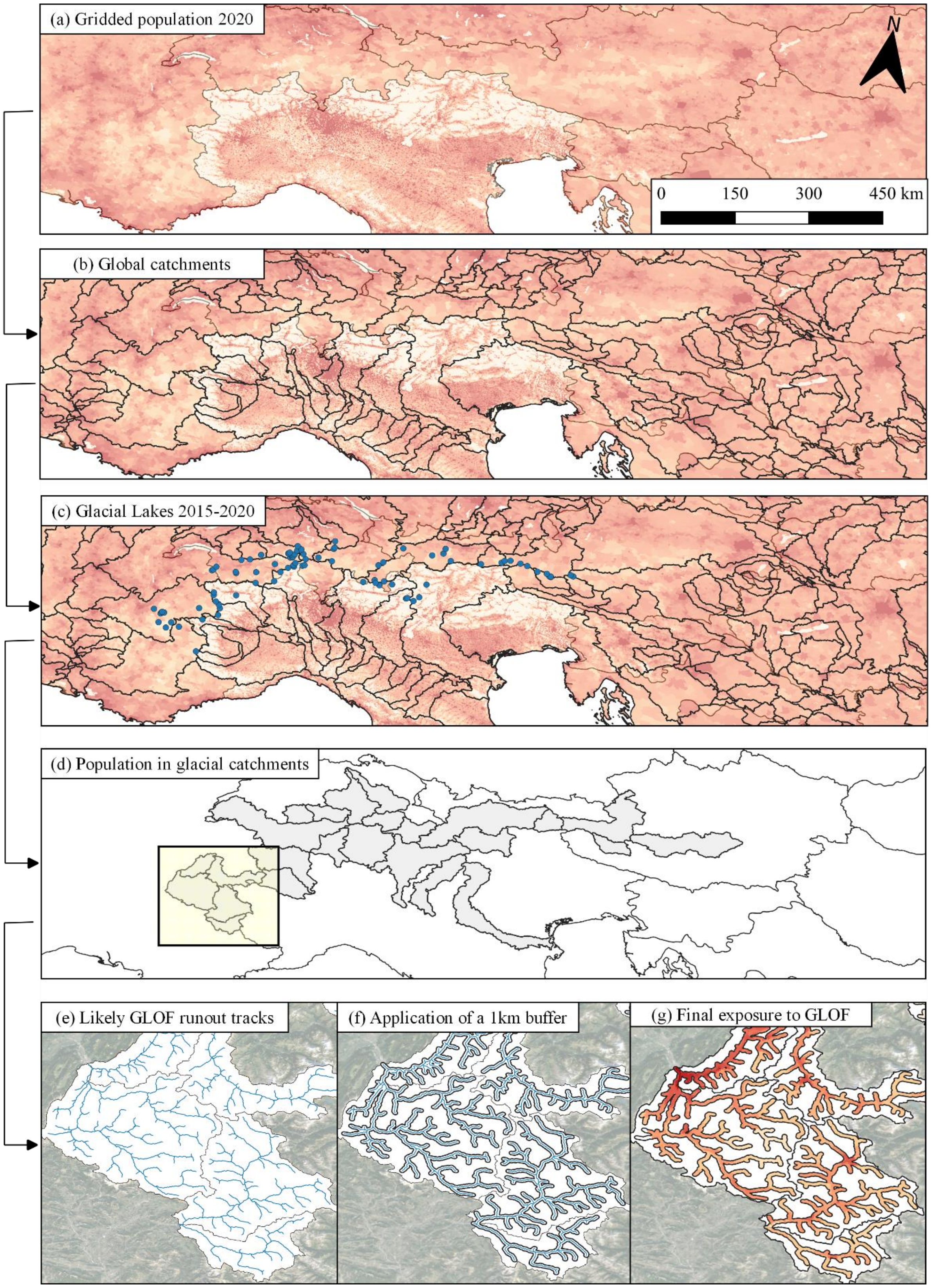

Supplementary figure 5: Extraction of exposed population. Workflow detailing the extraction of population exposure; a) global gridded population 2020, b) application of river basins, c) glacial lake shapefiles 2015-2018, d) extraction of glacial basins (grey), e) glacial-fed river channels as proxies for likely GLOF runout tracks up to 50 km from glacial lakes, f) application of 1 km buffer either side of river channels and h) final exposed population extracted. For reference, basins shown in panels e-g clockwise from the top are as follows; FRA40210090200, FRA40210090201, FRA40210030500, FRA40210040401 and FRA40210090101. Background of d-f Google Earth 2021.

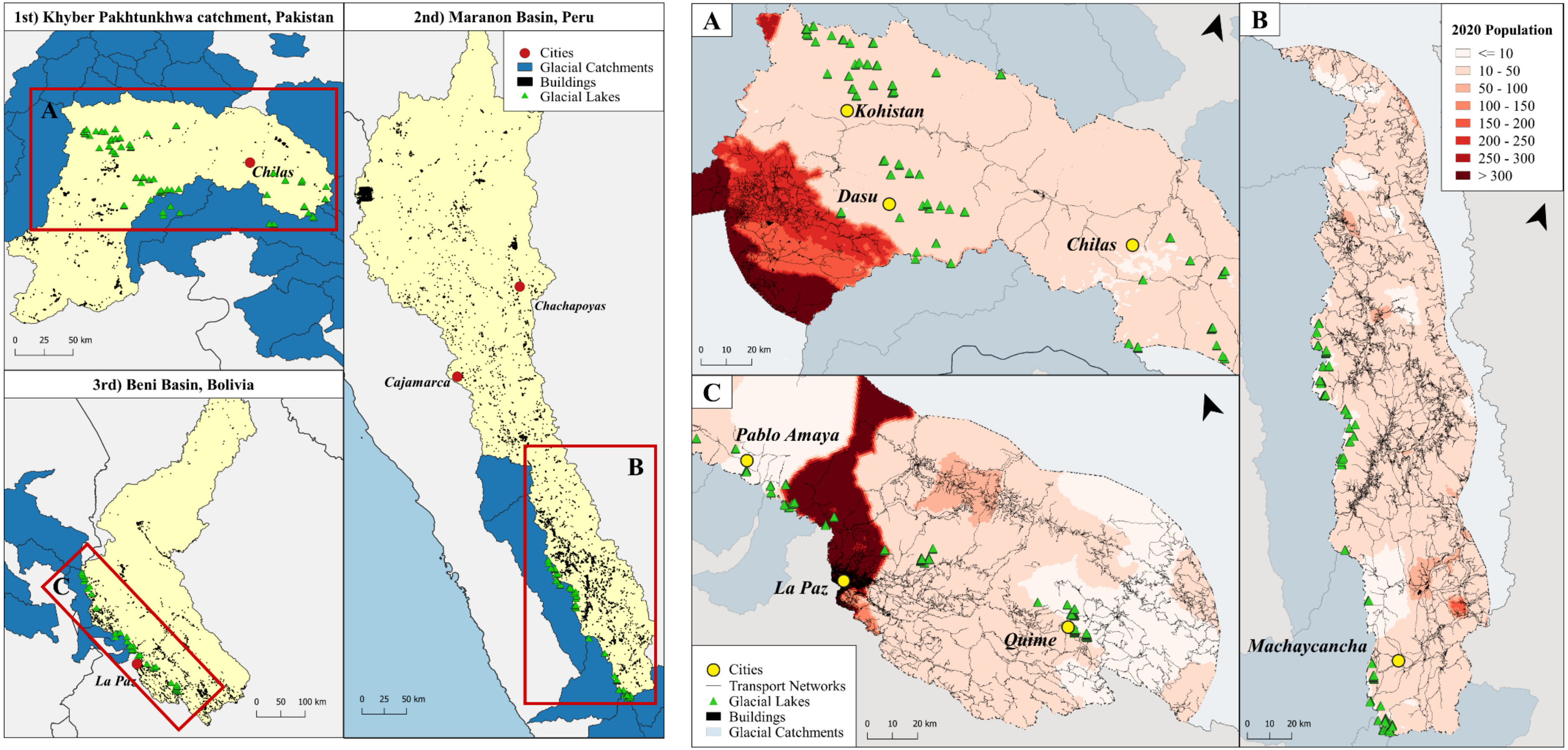

Supplementary figure 6: Top 3 most dangerous basins. Location of the three most dangerous glacial basins as of 2020; 1st - Khyber Pakhtunkhwa basin, Pakistan, 2nd - Santa basin, Peru and 3rd - Beni basin, Bolivia. Inset panels show areas of higher exposure within these basins. Key cities, transport networks (railways/roads) and buildings are shown alongside the location of glacial lakes as of 2020. Population as of 2020 is given.

Supplementary Table 1: Top 50 basins in terms of GLOF danger as of 2020. Countries are coloured according to mountain range, where; Alps = red, Andes = blue, HMA = green, PNW = purple and High Arctic and Outlying Countries = orange. *Very few of the basins included have clearly identifiable names within literature, and for some of the smaller basins the names vary between local populations and across languages. Thus, basin names are given where possible alongside basin ID numbers that can be used to identify others if needed.

Country	Catchment ID	Normalised Danger	Danger Rank	Catchment Name*
PAK	11411000000	1.47E-03	1	Khyber Pakhtunkhwa
PER	50432000000	3.43E-04	2	Santa
BOL	50411230301	3.40E-04	3	Beni
BTN	11501060201	2.61E-04	4	Punatsangchhu
IND	11501050200	1.75E-04	5	
CHL	51203030111	1.69E-04	6	
PAK	11410040300	1.60E-04	7	
ARG	51202010101	1.40E-04	8	
USA	30101130110	1.39E-04	9	
USA	30403131201	1.31E-04	10	
NOR	41001010201	1.10E-04	11	Glomma
PER	51502040200	9.47E-05	12	
PAK	11410040201	9.40E-05	13	
PAK	11406150100	7.95E-05	14	Sutlej
CHN	11504090000	7.78E-05	15	
NOR	41001010211	7.33E-05	16	Møre og Romsdal
IND	11406190000	7.17E-05	17	Jhelum
NPL	11504080301	6.83E-05	18	TamaKoshi
NPL	11504080500	6.55E-05	19	BhoteKoshi
NPL	11506070000	5.37E-05	20	Trishuli
CHE	40210100400	5.07E-05	21	Rhône
ITA	40208060200	4.44E-05	22	
CHL	51202050211	4.39E-05	23	
NPL	11506050100	4.07E-05	24	Marsyangdi
NPL	11504080101	3.95E-05	25	DudhKoshi
AFG	10907220101	3.92E-05	26	
TJK	10907330101	2.89E-05	27	Pyanj and Kyzylsu
IND	11406190100	2.83E-05	28	Chenab
CHL	51301020211	2.80E-05	29	
NPL	11504070100	2.75E-05	30	Tamor
NOR	41001050911	2.49E-05	31	Rogaland
PER	50426270600	2.25E-05	32	
ARG	51301060200	2.23E-05	33	Santa Cruz
IND	11406180100	2.16E-05	34	Indus
PER	50426221300	2.12E-05	35	
CHN	11002030201	1.80E-05	36	Heihe
CAN	30401070401	1.73E-05	37	
PER	51502030101	1.70E-05	38	
PER	50411231301	1.68E-05	39	
BTN	11501110301	1.65E-05	40	Drangmechhu
CHN	12517000000	1.65E-05	41	Jinsha
CHN	11002031101	1.52E-05	42	
BTN	11501110201	1.48E-05	43	Mangdechhu
NOR	41001051211	1.38E-05	44	
IND	11501050101	1.29E-05	45	
PER	51401160500	1.26E-05	46	
PER	51401160101	1.25E-05	47	
AUT	40515060000	1.21E-05	48	
ITA	40208140000	1.19E-05	49	
BTN	11501060500	1.11E-05	50	Amochhu

Supplementary Table 2: Values used to calculate the Social Vulnerability Index for the vulnerability proxy. Values are given as percentages of total population. Metrics in red increase vulnerability to GLOF, those in green reduce vulnerability to GLOF. Blank indicates no data available. Countries are coloured according to mountain range, where; Alps = red, Andes = blue, HMA = green, PNW = purple and High Arctic and Outlying Countries = orange.

Country ID	Population <5 yrs	Population >65 yrs	Illiterate Population	Unemployed Population	Literate Female Population	Urban population	Safe Drinking Water	Good Sanitation	Internet Users
AFG	14.8	2.6	57.0	11.1	13.2	25.8			13.5
ARG	8.4	11.2	1.0	9.8	59.2	92.0			71.8
AUT	4.9	19.4	1.0	4.7	100.0	58.5	99.0	97.0	87.7
BOL	10.3	7.3	7.5	3.5	53.1	69.8		23.0	43.8
BTN	8.3	6.1	33.5	6.8	23.3	41.6	36.0		48.1
CAN	5.3	17.6	1.0	5.6	100.0	81.5	99.0	82.0	91.0
CHL	6.2	11.9	3.6	7.1	77.8	87.6	99.0	77.0	82.3
CHN	5.4	11.5	3.2	4.3	76.0	60.3		72.0	54.3
COL	7.4	8.8	4.9	9.7	55.7	81.1	73.0	17.0	62.3
ECU	9.6	7.4	7.2	4.0	52.5	64.0	75.0	42.0	57.3
FRA	5.6	20.4	1.0	8.4	81.7	80.7	98.0	88.0	82.0
GEO	6.9	15.1	0.6	14.4	97.2	59.0	80.0	27.0	64.0
GRL	5.2	20.0	1.0	4.9	91.2	88.0	97.0	95.0	97.6
IND	8.5	6.4	25.8	5.4	27.7	34.5			34.5
ISL	6.1	15.2	1.0	2.8	100.0	93.9	100.0	82.0	99.0
ITA	3.9	23.0	0.8	9.9	75.9	70.7	95.0	96.0	74.7
KAZ	10.5	7.7	0.2	4.6	99.3	57.5	90.0		78.9
KGZ	12.0	4.6	0.4	6.3	99.1	36.6	68.0		38.0
MNG	11.6	4.2	1.6	6.0	91.5	68.5	24.0		23.7
NOR	5.2	17.3	1.0	3.3	95.4	82.6	98.0	76.0	96.5
NPL	9.5	5.8	32.1	1.4	29.3	20.2	27.0		34.0
NZL	6.3	16.0	1.0	4.1	97.4	86.6	100.0	89.0	90.8
PAK	12.8	4.3	40.3	4.5	27.6	36.9	35.0		15.5
PER	8.6	8.4	5.6	3.3	58.9	78.1	50.0	43.0	52.5
RUS	6.4	15.1	0.3	4.6	96.3	74.6	76.0	61.0	80.9
SWE	6.0	20.2	1.0	6.5	89.3	87.7	100.0	93.0	92.1
CHE	5.2	18.8	1.0	4.6	95.6	73.8	95.0	100.0	89.7
TJK	14.4	3.1	0.2	11.0	93.3	27.3	48.0		22.0
USA	6.0	16.2	1.0	3.7	96.1	82.5	99.0	90.0	87.3
UZB	10.4	1.3	0.0	5.9	99.9	50.4	44.0		52.3
